# Haplotype assignment of longitudinal viral deep sequencing data using covariation of variant frequencies

**DOI:** 10.1093/ve/veac093

**Published:** 2022-10-06

**Authors:** Cristina Venturini, Juanita Pang, Asif U Tamuri, Sunando Roy, Claire Atkinson, Paul Griffiths, Judith Breuer, Richard A Goldstein

**Affiliations:** Infection, Immunity, Inflammation, Institute of Child Health, University College London, London WC1E 6BT, UK; Division of Infection and Immunity, University College London, London WC1E 6BT, UK; Research IT Services, University College London, London WC1E 6BT, UK; Infection, Immunity, Inflammation, Institute of Child Health, University College London, London WC1E 6BT, UK; Institute for Immunity and Transplantation, University College London, London NW3 2PP, UK; Institute for Immunity and Transplantation, University College London, London NW3 2PP, UK; Infection, Immunity, Inflammation, Institute of Child Health, University College London, London WC1E 6BT, UK; Great Ormond Street Hospital for Children, London WC1N 3JH, UK; Division of Infection and Immunity, University College London, London WC1E 6BT, UK; Infection, Immunity, Inflammation, Institute of Child Health, University College London, London WC1E 6BT, UK

**Keywords:** haplotype reconstruction, next-generation sequencing, human cytomegalovirus, norovirus

## Abstract

Longitudinal deep sequencing of viruses can provide detailed information about intra-host evolutionary dynamics including how viruses interact with and transmit between hosts. Many analyses require haplotype reconstruction, identifying which variants are co-located on the same genomic element. Most current methods to perform this reconstruction are based on a high density of variants and cannot perform this reconstruction for slowly evolving viruses. We present a new approach, HaROLD (HAplotype Reconstruction Of Longitudinal Deep sequencing data), which performs this reconstruction based on identifying co-varying variant frequencies using a probabilistic framework. We illustrate HaROLD on both RNA and DNA viruses with synthetic Illumina paired read data created from mixed human cytomegalovirus (HCMV) and norovirus genomes, and clinical datasets of HCMV and norovirus samples, demonstrating high accuracy, especially when longitudinal samples are available.

## Introduction

Next-generation sequencing (NGS) of virus populations derived from medical and biological samples can deepen our understanding of virus biology, pathogen evolution, host–pathogen interactions, transmission dynamics, and the development of drug resistance (Houldcroft, Beale, and Breuer 2017; Leung et al. 2017; Moncla et al. 2017). Virus genomes are smaller than bacterial and eukaryotic genomes, but are still larger than Illumina NGS reads. Detailed analyses often require determining which variants are found together in the same genome or genomic segment, a process known as haplotype reconstruction. This is commonly performed by identifying variants at sites that are close enough to be found on the same reads. If these variants are sufficiently dense, co-occurring variants across the genome can be ‘stitched together’, resulting in the determination of whole-genome haplotypes (Posada-Cespedes, Seifert, and Beerenwinkel 2017). Several computer programs have been developed over the last decade using this approach to reconstruct haplotypes from NGS data, including PredictHaplo (Prabhakaran et al. 2014) and CliqueSNV (Knyazev et al. 2021). A recent work by Eliseev and colleagues (Eliseev et al. 2020) benchmarked several of these tools and found that PredictHaplo and CliqueSNV outperformed the others. However, all these tools have been created and tested with small- and fast-evolving viruses such human immunodeficiency virus 1 (HIV-1) and hepatitits C virus (HCV). Unfortunately, viruses such as human cytomegalovirus (HCMV; species *Human betaherpesvirus 5*) can have long regions with few segregating sites, making it impossible to connect variants that span these regions.

There is increased focus on monitoring intra-host evolutionary dynamics using longitudinal sequencing, where samples are obtained from a single patient at multiple time points. Selection and drift result in changes in the relative frequencies of the haplotypes and thus in the frequencies of the variants that they contain. In such cases, we can use covariation of variant frequencies to provide an additional source of information for haplotype reconstruction, even when these variants are far apart in the genome. To take advantage of data from longitudinal sampling and include bigger recombining viruses, such as herpesviruses, we created a new method for reconstructing whole-genome haplotypes from longitudinal sequence data (HAplotype Reconstruction Of Longitudinal Deep sequencing data, HaROLD). Few other tools have been developed that use frequency data to reconstruct haplotypes, such as EVORhA (Pulido-Tamayo et al. 2015), which was specifically developed for bacteria. CliqueSNV, which has similarities to the first stage described here, showed good accuracy for haplotype frequencies in simulated data, but lower accuracy for haplotype reconstruction in comparison to HaROLD (Pelizzola et al. 2021).

Here, we describe HaROLD and compare its performance with CliqueSNV, PredictHaplo, and EVORhA. These comparisons were performed using synthetic NGS data obtained by simulating longitudinal sampling for two different types of viruses studied in our laboratory (lab): norovirus (species *Norovirus*), a highly diverse RNA virus, and HCMV, a large (235k bp), slowly evolving DNA virus. We also illustrate how HaROLD works compared to other methods with real data from two immunocompromised patients; one infected with HCMV and one with Norovirus. Application of this approach to real data from mixed-infected HCMV patients has been presented previously (Cudini et al. 2019; Pang et al. 2020).

## Results

We consider that we have sets of reads from a number of samples analysed using NGS, where all of the samples share a common set of related haplotypes. These may, for instance, represent a series of virus samples that have been extracted from a single patient at various time points (longitudinal samples). Note that the number of samples can be as small as one, and each sample does not necessarily contain every haplotype (the frequency of a haplotype in some samples may be zero). We are interested in determining the sequences of the haplotypes and their frequencies in each of the samples based on the observed reads.

HaROLD performs the following steps:

1. Initial estimation of haplotype sequences and frequencies taking advantage of covariation of variant frequencies.
2. Refinement of haplotypes through analysis of observed reads, incorporating information from co-occurring variants.

These steps are described briefly here and in more detail in the Methods and Supplementary Materials.

### Initial estimation

In this initial step, we assume that the samples contain a common set of identical haplotypes but in differing proportions. In order to make an initial estimation of the haplotypes, we employ a statistical model that describes the observed sequence data consisting of (1) a set of haplotype frequencies, representing the frequency of each of the haplotypes in each sample, and (2) a distribution of sequencing error rates, represented by a Dirichlet distribution. The statistical model does not include the sequences of the haplotypes. Instead, we consider these sequences, as well as the rates for specific sequencing errors at specific sites, to represent unknown ‘nuisance parameters’. In this initial stage, we consider each site in the genome separately. Although we lose information about the co-occurrence of variants at different sites along the reads, this simplification allows us to avoid a complicated and costly *exploration* of the space of possible haplotype sequences in favour of a simple *sum* over the ${4^H}$ ways of assigning bases to each site in the $H$haplotypes.

We optimise the haplotype frequencies and error rate parameters to maximise the likelihood of the data, where the calculation of the likelihood involves an explicit summation over the sequences of the haplotypes and an integration over the error rates. (This is similar to other hybrid maximum likelihood/Bayesian approaches, such as in phylogenetics where the phylogenetic tree is optimised based on a likelihood calculation that sums over all possible combinations of substitutions.)

Once we have derived the optimal haplotype frequencies and the error rate parameters, we can calculate the posterior probability that each possible base occurs at each site in each of the haplotypes. This provides a probabilistic reconstruction of the haplotypes, indicating the appropriate degree of confidence one should have about the haplotype reconstruction of each site. Whenever this probability is sufficiently high, we can assign a specific base to that haplotype.

Although we use standard quality controls to distinguish spurious reads, we do not attempt to distinguish reliable and erroneous bases based on, for instance, number or frequency of observation. Rather, we explicitly model the probability that a specific base is observed, either correctly or erroneously. The estimation of this probability depends on a characterisation of the error rate, which may depend on the true base, the observed base, the location in the alignment, the direction of the read, and the sample in which this read was present. Rather than assuming a fixed error rate, we model true-base-, observed-base-, location-, direction-, sample-specific error rates as independent draws from a Dirichlet distribution with parameters ${\alpha _0}$ and ${\alpha _\epsilon }$, which are optimised during this initial step. The representation of the distribution of error rates as a Dirichlet distribution allows a closed-form integration over error rates.

This procedure is repeated for a range of different numbers of haplotypes. Increasing the number of haplotypes increases the number of ways of assigning bases to haplotypes, decreasing the prior probability of any given assignment. As is common in Bayesian methods, this results in the log likelihood decreasing as the number of haplotypes increases beyond that necessary to represent the data. We choose the number of haplotypes that maximises the log likelihood of the read data.

### Refinement process

The initial estimation step previously assumes that the set of haplotype sequences are identical for the various samples, neglecting mutations that might occur between samples. It also ignores the information that forms the basis of most haplotype reconstruction methods, the presence of multiple variants on the same read. The next step is to relax these assumptions and use variant co-localisation to refine the haplotypes.

**Figure 1.**
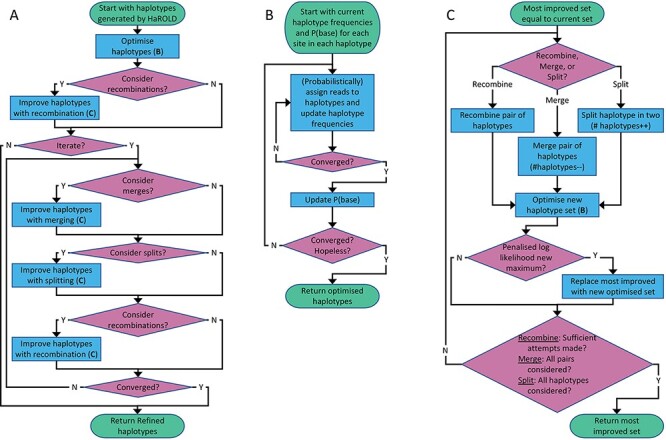
Flowchart of refinement process. (A) Overall process. (B) Subprocess for haplotype optimisation. (C) Subprocess for considering recombination, merging, and splitting; merging decreases the number of haplotypes by one, while splitting increases this number by one. Support for these three operations is evaluated by considering a penalised log likelihood equal to −0.5 times the Akaike Information Criterion.

In this refinement step (Fig. 1), each sample is analysed individually. We start with the estimated frequencies of each haplotype in this sample and the a posteriori probability of each base at each site in each haplotype, as estimated in the initial step described previously. Based on these parameters, each read is assigned probabilistically to each of the various haplotypes. The number of reads assigned to each haplotype is used to adjust the frequencies of that haplotype. The reads are then reassigned until the haplotype frequencies have converged. The resulting assigned reads are then used to update the probability of the bases found in each site in all the reads assigned to each haplotype. The reads are then reassigned based on these adjusted probabilities, and this procedure repeated until convergence. These two steps—estimation of haplotype frequencies and estimation of base probabilities—are then alternated until convergence. We also calculate the log likelihood of the read data given these parameter values, as well as a penalised log likelihood from which the number of adjustable parameters has been subtracted, equivalent to −0.5 times the Akaike information criterion (Akaike 1998).

If requested by the users, a number of structural modifications of the haplotypes are considered. These include (1) recombination of two haplotypes, where corresponding regions of the haplotype sequences are swapped, (2) gene conversion, where a region of one haplotype sequence is overwritten by the corresponding region of a different haplotype sequence, (3) merging of two haplotypes into a single new haplotype, reducing the total number of haplotypes by one, and (4) dividing a single haplotype into two new haplotypes, increasing the total number of haplotypes by one. After each of these modifications, the haplotype frequencies and base probabilities are readjusted as described previously, and the modification rejected or accepted based on whether it results in a decrease or increase in the penalised log likelihood. These modifications result in a final set of haplotypes whose size represents the number of haplotypes that can be justified, based on information theory, by the sequence data.

The output of the program includes, for each sample, the frequencies of the haplotypes as well as the probabilities of each of the bases at each of the sites in the haplotypes. When this probability is over a user-defined value, the site can be assigned to a specific base.

### Simulation results

To evaluate the ability of HaROLD to reconstruct haplotypes and estimate the relative haplotype frequencies, we created eight synthetic sequence datasets (four for norovirus and four for HCMV), each consisting of a set of sequential longitudinal samples drawn from differing mixtures of whole-genome sequences from GenBank, as summarised in Tables 1 and 2. The norovirus dataset (Table 1) each consisted of five longitudinal samples of between two and four haplotypes with varying degrees of similarity (total number of norovirus samples = 20). The HCMV datasets (Table 2) were constructed in a similar manner, each with three longitudinal samples (total number of HCMV samples = 12) constructed from two or three haplotypes.

**Table 1.** Summary of the longitudinal norovirus synthetic datasets used to test the accuracy of the haplotype reconstruction methods. Four synthetic datasets with five samples each were created for norovirus by mixing GenBank sequences for a total of twenty samples.

Norovirus		
Set	Sample composition	Similarity between haplotypes (percentage identity)
Two haplotypes Low similarity Five time points	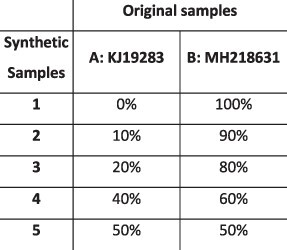	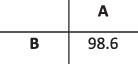
Two haplotypes High similarity Five time points	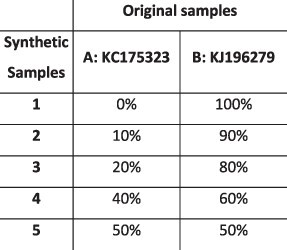	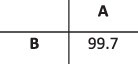
Three haplotypes Five time points	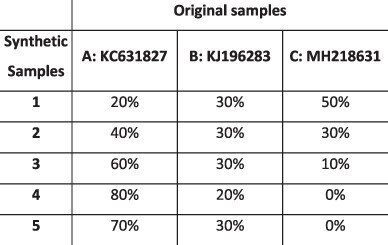	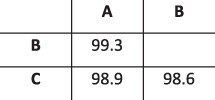
Four haplotypes Five time points	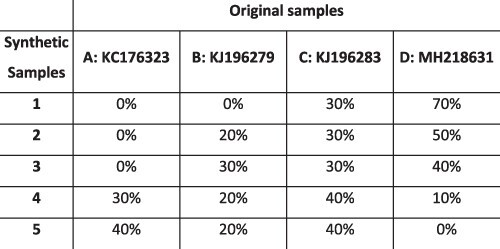	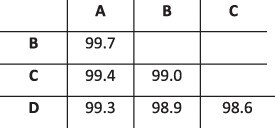

**Table 2.** Summary of the longitudinal human cytomegalovirus synthetic datasets used to test the accuracy of the haplotype reconstruction methods. Four synthetic datasets with three samples each were created for HCMV by mixing GenBank sequences for a total of twelve samples.

HCMV		
Set	Sample composition	Similarity between haplotypes (Percentage identity)
Two haplotypes Low similarity Three time points	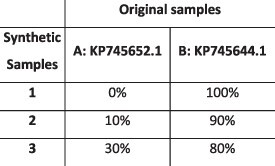	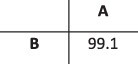
Two haplotypes High similarity Three time points	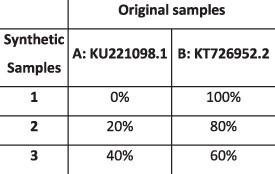	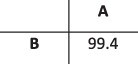
Three haplotypes Low similarity Three time points	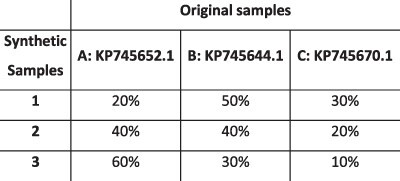	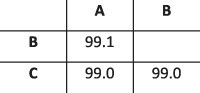
Three haplotypes High similarity Three time points	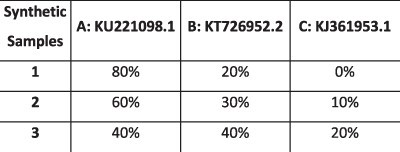	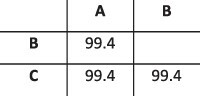

#### Reconstruction of haplotype sequences and frequency

**Figure 2.**
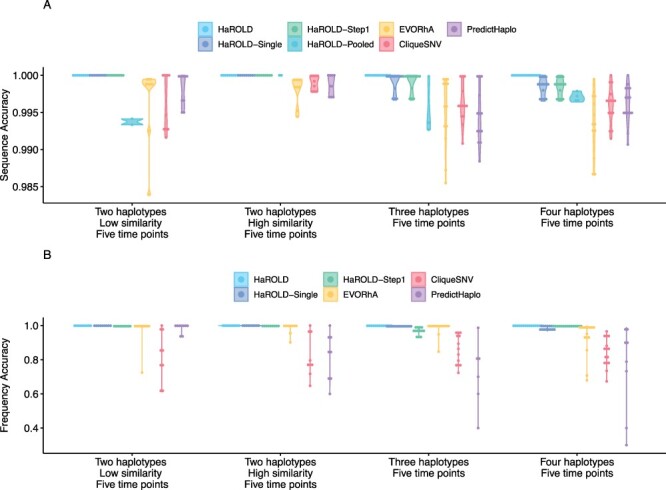
Violin plots showing the accuracy of haplotype reconstruction in the norovirus test datasets. (A) The accuracy of reconstructed sequences (pairwise identity between the actual sequences and reconstructed sequences). (B) The accuracy of estimated frequencies. Colours indicate different haplotype reconstruction methods. Each dot represents a sequence from each sample.

**Figure 3.**
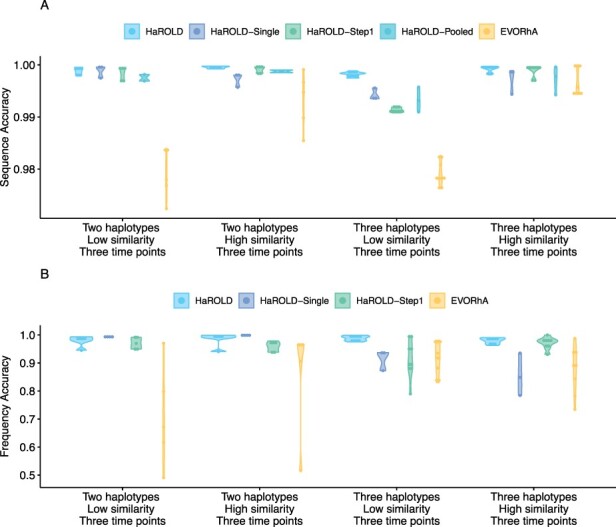
Violin plots showing the accuracy of haplotype reconstruction in the HCMV test set. (A) The accuracy of reconstructed sequence (pairwise identity between the actual sequences and reconstructed sequences). (B) The accuracy of estimated frequencies. Colours indicate different haplotype reconstruction methods. Each dot represents a sequence from each sample.

The performance of HaROLD on the synthetic data is represented in Figs 2 and 3 (in sky blue). Performance was calculated as (1) the proportion of sites that are identical between GenBank sequences and the reconstructed haplotypes and (2) the difference between the real haplotypes frequencies and frequencies calculated by HaROLD. With the norovirus data, the reconstructed haplotypes were identical to the GenBank sequences (accuracy 100 per cent in all four datasets) (Fig. 2A). The haplotype frequencies estimated by HaROLD were also highly accurate, with differences between the actual and estimated frequencies less than 0.002 (Fig. 2B). Excellent results were also obtained with the synthetic data derived from HCMV; the reconstructed haplotypes were highly similar to the original sequences (similarity > 0.997) (Fig. 3A) with differences between the actual and computed haplotype frequencies less than 0.06 (Fig. 3B). HaROLD computational time (in an high performance computing (HPC) node with a maximum of 50 GB memory) was dependent on the number of haplotypes, average read depth, and genome length and varied from 40 to 226 s for norovirus and 35 to 39 min for HCMV (Table 3). This can be longer in some cases, generally dominated by the estimation of the error rate parameters. The calculations can, correspondingly, be greatly sped up if these parameters are estimated and fixed.

**Table 3.** Computational time for HaROLD and other software for analysing norovirus and HCMV datasets.

Norovirus				
	Two haplotypes Low similarity Five time points	Two haplotypes High similarity Five time points	Three haplotypes Five time points	Four haplotypes Five time points
Harold	40 s	1 min 4 s	48 s	3 min 46 s
CliqueSNV	13 min 27 s	20 min 24 s	7 min 7 s	13 min 43 s
PredictHaplo	5 h 17 min	6 h 27 min	4 h 40 min	5 h 4 min
EVORhA	18 min	19 min	16 min	20 min
HCMV				
	Two haplotypes Low similarity Three time points	Two haplotypes High similarity Three time points	Three haplotypes Low similarity Three time points	Three haplotypes High similarity Three time points
Harold	39 min	36 min 28 s	25 min	26 min
EVORhA	6 min	5 min	8 min	6 min

#### Utility of longitudinal sampling

In contrast to most methods for haplotype reconstruction, HaROLD is formulated to take advantage of the availability of multiple longitudinal samples. To evaluate the importance of these longitudinal samples, we used HaROLD to reconstruct the haplotypes in our synthetic datasets without using this additional information. We compared performance of HaROLD using (1) single samples run independently (HaROLD-Single) and (2) longitudinal samples pooled together in one sample (HaROLD-Pooled). In both norovirus and HCMV, the performance of HaROLD-Single and HaROLD-Pooled was high, varying from 0.99 to 1 similarity to the GenBank sequences, decreasing with the increase of number of haplotypes (Figs 2A and 3A in blue and in turquoise, respectively). For HaROLD-Single, estimated frequencies were 93–100 per cent similar to actual frequencies in norovirus and 78–99 per cent for HCMV, not as accurate as when HaROLD was run as designed with longitudinal samples (Figs 2B and 3B). In general, the performance of HaROLD on HaROLD-Single and HaROLD-Pooled was not as accurate as when longitudinal data were used, highlighting the advantage of using serial sampling. Even so, the accuracies of the haplotype reconstructions were generally quite high, especially for the shorter norovirus sequences and when there were relatively few haplotypes.

#### Refinement step

As described previously and in more detail in Methods, HaROLD uses two different steps to reconstruct the final haplotypes and frequency. We evaluated the performance and utility of the additional refinement step by comparing results from HaROLD and HaROLD without this additional refinement step (HaROLD-Step 1). In both Norovirus and HCMV, the reconstructed haplotypes for HaROLD-Step 1 were accurate, varying from 0.996 to 1 (Norovirus) and 0.99 to 0.999 (HCMV), decreasing as number of haplotypes increase (Figs 2A and 3A in green). Estimated frequencies were accurate (Norovirus 93–100 per cent; HCMV 79–99 per cent) (Figs 2B and 3B in green). However, both datasets achieved a lower accuracy in HaROLD-Step 1 as compared to results obtained with HaROLD (both steps). The difference is larger in the HCMV dataset, especially in the dataset with three haplotypes with low similarity.

#### Comparison with other methods

We compared the performance of HaROLD with two haplotype reconstruction techniques reviewed by Eliseev et al. (2020), namely CliqueSNV and PredictHaplo; these two methods performed well in terms of accuracy in their HIV validation. CliqueSNV is a reference-based method to reconstruct haplotypes from NGS short reads data, which constructs an allele graph based on linkage between variants and identifies true viral variants by merging cliques of that graph via combinatorial optimization techniques (Knyazev et al. 2021). PredictHaplo implements a fully probabilistic approach to quasispecies reconstruction. Given a set of aligned reads, it uses a Bayesian mixture model with a Dirichlet process prior to estimate the unknown number of underlying haplotypes (Prabhakaran et al. 2014). We also added a comparison with a third method, EVORhA (Pulido-Tamayo et al. 2015), that was developed for bacterial haplotype reconstruction and combined phasing information in regions of overlapping reads with the estimated frequencies of inferred local haplotypes. This method was chosen because it is one of the few other haplotype reconstruction methods which also considers variant frequencies. We ran these three methods using default parameters unless otherwise stated. All analyses were run on an HPC node with a maximum of 48 h and 50 GB of memory. In both the HCMV and norovirus datasets, EVORhA generally estimated a larger number of haplotypes than present in the sample (ranging from 1 to 5 additional haplotypes) and consistently yielded haplotypes that most resembled the input reference sequence used for mapping. The sequence accuracy ranged from 0.972 to 0.999 for HCMV (Fig. 3A in yellow) and from 0.983 to 0.999 for Norovirus (Fig. 2A in yellow), consistently lower than HaROLD. The performance of EVORhA in estimating the relative haplotype frequencies was uneven and overall worse compared to HaROLD (Figs 2B and 3B). On the norovirus datasets, CliqueSNV yielded more accurate haplotype sequences than EVORhA; frequency accuracy was, however, uneven (Fig. 2A and B in red). PredictHaplo performed similarly to CliqueSNV (sequence accuracy from 0.988 to 1, Fig. 2A in purple) and again frequency accuracy was uneven, especially with four haplotypes (Fig. 2B). HaROLD consistently outperformed these other techniques in both sequence and frequency accuracy, even when single samples were run independently (as explained in the previous section *Utility of longitudinal sampling*) (Fig. 2). We were not able to analyse the HCMV datasets using CliqueSNV and PredictHaplo due to memory constraints; both programs were developed for smaller RNA viruses such as HIV and were not able to analyse a genome as large as ∼250k bp with available computational resources. HaROLD was generally faster than the other methods for the norovirus datasets, although EVORhA was faster for the HCMV datasets where the average read depth was low (Table 3).

#### Diversity calculation

**Figure 4.**
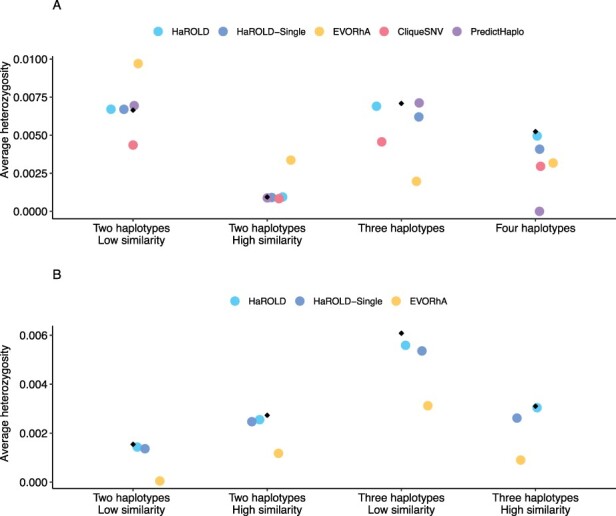
Accuracy of sample diversity (measured as average heterozygosity) estimations based on reconstructed haplotypes for norovirus dataset (A) and HCMV dataset (B). Average heterozygosity was estimated for one sample for each set for both norovirus (Set1—Sample4, Set2—Sample3, Set3—Sample1, Set4—Sample4) and HCMV (Set1—sample2, Set2—Sample3, Set3—Sample2, Set4—Sample2) True sequence heterozygosity is shown with black diamonds.

As an example of the consequences of the different reconstruction accuracies on downstream analyses, we estimated the average heterozygosity of the various samples based on the reconstructed haplotypes, as shown in Fig. 4. The haplotypes generated by HaROLD produced accurate estimates of the average heterozygosity, especially in the longitudinal dataset. PredictHaplo generally produced accurate heterozygosity in norovirus, albeit the accuracy decreased when four haplotypes were present. CliqueSNV underestimated heterozygosity in almost all conditions, except for when we had two norovirus haplotypes that were very similar. Finally, EVORhA underestimated heterozygosity in both HCMV and norovirus in almost all datasets, except in norovirus with two haplotypes.

### Application to real data

We applied our approach to two datasets: an unpublished dataset of HCMV samples from kidney/liver recipients where there was contamination from a laboratory strain and a dataset including longitudinal samples of norovirus from an immunocompromised patient as described by Ruis et al. (2018).

#### HCMV contamination dataset

**Figure 5.**
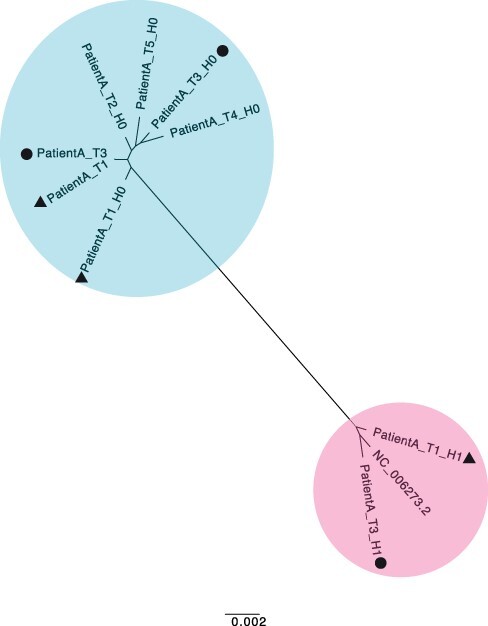
Maximum likelihood phylogenetic tree of reconstructed haplotypes and consensus sequences for the patient infected with HCMV. Samples of HCMV were taken from PatientA at five time points (T1–T5); reconstructed haplotypes are indicated with H0 and H1. The two haplotypes from the original sample and the consensus sequence from the re-sequenced sample for time point T1 are indicated with a triangle and the corresponding sequences for T3 with a circle. The two viral populations/strains present in the tree are coloured in blue and pink.

During analysis of a set of five longitudinal samples taken from a 42-year-old patient following a liver transplant (PatientA, T1–T5), we observed a high degree of within-host diversity in two of the samples. HaROLD was used to look for the presence of distinct haplotypes, yielding two haplotypes for samples T1 and T3 and only a single haplotype for the other three samples. The second haplotype in T1 and T3 was nearly identical to the Merlin laboratory strain (NC.006273.2), a strain present in the sequencing lab. This was subsequently identified as a sample contaminant. Following discovery of the contamination, these two samples were re-sequenced without the contaminant, providing a real-world scenario for validation of HaROLD with typical Illumina sequencing errors and uneven read depth and coverage. We built a maximum likelihood phylogenetic tree including the haplotypes reconstructed by HaROLD (indicated as H0 and H1) and the consensus sequences obtained from re-sequenced samples T1 and T3 (Fig. 5). The two haplotypes (H0 and H1) for each of the contaminated samples clearly clustered separately in the phylogenetic tree: H0 clustered together with the consensus sequences of T1 and T3 (blue cluster) whereas H1 clustered with Merlin GenBank sequence (pink cluster); the two clusters were approximately 98.5 per cent similar, with the 4,000+ differences largely in the ‘hypervariable genes’ (Suárez et al. 2019). We directly compared the HaROLD haplotypes and the consensus sequence for both samples. Sample T1 resulted in only seven differences between patientA_T1_H0 and the consensus sequence patientA_T1; these nucleotide differences clustered in *IRS1* gene which is a region full of repeats in HCMV. Sample T3 showed 312 SNP differences between patientA_T3_H0 and patientA_T,3 and again, these were in *IRS1* and *TRS1* genes, which contained repeats and are difficult to assemble and align.

#### Norovirus dataset

**Figure 6.**
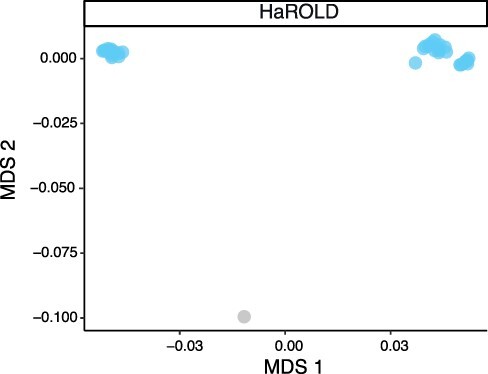
Multidimensional scaling (MDS) of HaROLD reconstructed haplotypes for patient infected with norovirus. Pairwise differences between haplotypes were calculated and used for MDS clustering. The plot shows the first two components. Reference GenBank strain is coloured in grey.

**Figure 7.**
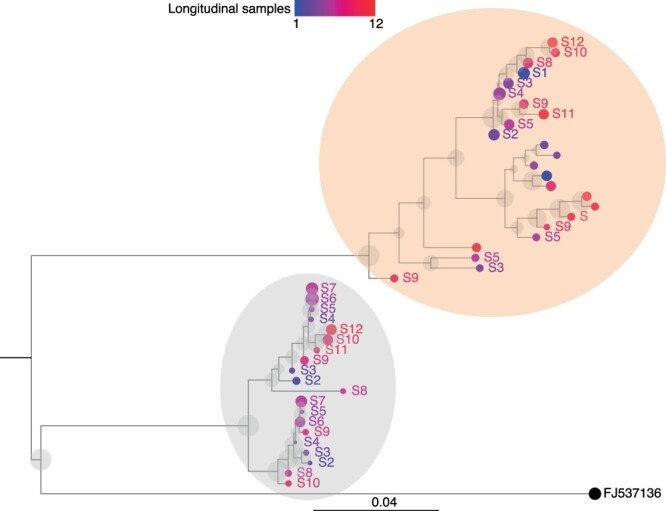
Maximum likelihood phylogenetic tree of HaROLD reconstructed haplotypes for patient infected with norovirus. Twelve samples were available for this patient (S1–S12) and were coloured differently using a continuous scale representing time (from blue S1 to red S12). The tips’ size indicates the frequency of the haplotype. The black sequence is the GenBank strain used for mapping (tip size set as 50 per cent frequency). Grey transparent circles represent the bootstrap values (1,000 bootstraps). Two viral populations were identified represented in orange and grey transparent circles.

**Figure 8.**
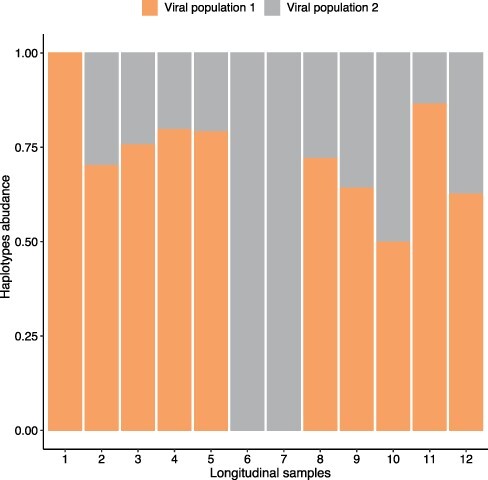
Bar plot of estimated abundances overtime of HaROLD reconstructed haplotypes for patient infected with norovirus. Two viral populations were present coloured in orange and grey, matching the colours on the maximum likelihood phylogenetic tree (Fig. 7).

We used HaROLD to analyse norovirus deep sequencing samples from an immunocompromised 48-year-old patient with chronic norovirus infection previously published (Ruis et al. 2018) (Pang J et al., manuscript in preparation). We collected twelve longitudinal samples over almost a year during which time the patient was treated with antiviral drug Favipiravir. The patient showed symptomatic response to Favipiravir treatment, and the phylogenetic analysis showed evidence for selective pressure in the infecting norovirus population. To better understand whether and how different viral populations evolved overtime and in response to treatment, we reconstructed haplotypes from all samples using HaROLD. Each sample yielded 2–5 haplotypes which we used to build a multiple sequences alignment together with the closest GenBank reference sequence (FJ537136). Analysis of pairwise genetic distances showed a clear bimodal distribution (Supplementary Fig. S1) with two main clusters observed with multidimensional scaling (Fig. 6). The two clades were also present in the maximum likelihood phylogenetic tree (Fig. 7). The first viral cluster (orange) was present since the first time point and was dominant in almost all samples (Fig. 8). When the patient received extensive treatment with Favipiravir, a second viral population (grey) appeared and became the dominant viral strain at time points 6 and 7.

We then compared these results with those obtained using other haplotype reconstruction methods. PredictHaplo generally gave similar results compared to HaROLD: it generated 3–8 haplotypes for each sample which generally clustered in two main viral populations which, however, were not as clearly distinct as for HaROLD, with each cluster divided into two sub-clusters (Supplementary Figs S2–S4). Even though PredictHaplo did perform similarly to HaROLD, we encountered computational issues due to time and memory limits; it did not finish on five out of twelve samples (HPC node with 50 GB and 14-day time limit). Both CliqueSNV and EVORhA yielded many low-frequency haplotypes (EVORhA 2–10, CliqueSNV 4–8) that tended to form diffuse clusters or were similar to the reference sequence (Supplementary Figs S2, S3, S5, and S6), which did not give information about the evolution of viral populations over time. In addition, when sequences from all methods were compared together, EVORhA haplotypes were genetically dissimilar from haplotypes obtained with other methods (Supplementary Fig. S3).

## Discussion

Majority of methods for reconstructing haplotypes rely on reads that contain multiple polymorphic sites and thus require a sufficiently density of polymorphic sites so that the distances between such sites are closer together than the read length (Prabhakaran et al. 2014; Knyazev et al. 2021). This approach can be used, for example, with HIV-1 and HCV, two small- and fast-evolving viruses, frequently used in testing haplotype reconstruction’s methods. However, this is not always the case, especially for viruses such as HCMV where much of the observed sequence diversity is confined to short intervals. Even when there is copious variation, there may be closely related haplotypes where the haplotype-defining variants are separated by distances greater than the read length, making it difficult to assign these variants correctly to the otherwise similar haplotypes. HaROLD was motivated by the increasing availability of multiple samples that are likely to share closely related haplotypes, such as longitudinal studies of within-host evolution or samples from an outbreak cluster. Under such conditions, variant frequencies can provide an important additional source of information for making accurate haplotype reconstructions (Pelizzola et al. 2021). Notably, HaROLD generates haplotypes as accurate or more accurate than other tested methods even when multiple samples were not available. This greater accuracy was achieved with significantly less computing power and memory than the other methods we used for comparison, allowing rapid analysis of sequence data. It is important to note that both PredictHaplo and CliqueSNV were not able to produce results for HCMV at all, due to the size of the virus, showing that these programs would be impractical for bigger microbes such as herpes viruses and bacteria.

Even in a small virus such as norovirus, PredictHaplo encountered computational issues for 5/12 samples due to time and memory limits (HPC node with 50 GB and 14-day time limit).

Even if HaROLD was created to deal with big double-strand DNA viruses, it performed well with RNA viruses, such as norovirus. In such context, it is difficult to determine how many haplotypes there are in the sample, even with perfect information. One could consider every unique sequence in the sample as a different haplotype, but in this case, the number of haplotypes would generally be so large as to make any further analysis impractical. Alternatively, one could consider haplotypes as representing clusters of closely related sequences that do not need to be all identical. In this case, there is some flexibility in how one defines the term ‘closely related’. HaROLD is generally conservative about the number of haplotypes. In particular, the refinement method does not add an additional haplotype unless the improvement in the log likelihood is sufficient to justify the resulting increase in the number of parameters. The resulting haplotypes then include some amount of variation, which is provided as output to the user. In particular, the output reports the probability that a sequence belonging to a haplotype would have any of the four bases found in each site. When these probabilities are sufficiently definitive, a base is assigned in the multiple sequence alignment. An ambiguous base is presented when a definitive assignment cannot be made.

We described the performance of HaROLD in the analysis of synthetic datasets, as well as its use for two real-data examples, one for HCMV and one for Norovirus. In the HCMV example, HaROLD was able to reconstruct the patient’s sequence and the lab strain contaminated sequence with high accuracy. HaROLD was successfully used to detect and confirm the contamination in the first place and then reconstruct the ‘real’ sequence allowing us to use a sample which would have been discarded otherwise. This was a lab-created ‘mixed infection’ and no different from a real situation where a liver recipient may have a superinfection from reactivation of a HCMV strain already present and a new infection from a HCMV-positive donor following transplant. In the second example, we showed longitudinal samples from an immunocompromised patient chronically infected with norovirus. In this patient, we were able to distinguish two main viral populations with one selected during the drug treatment. EVORhA (Pulido-Tamayo et al. 2015) and CliqueSNV (Knyazev et al. 2021) generated larger numbers of haplotypes that either clustered by sample or very close to the reference used for mapping. PredictHaplo (Prabhakaran et al. 2014) performed similarly to HaROLD although it often did not converge within a reasonable timescale. In these examples, we illustrated how a precise haplotype reconstruction can be useful in determining the likelihood of mixed infection and/or how the viral populations respond to treatment and evolve over time.

In a previous paper, we described another application on real clinical data for HCMV (Cudini et al. 2019; Pang et al. 2020), where HaROLD was able to reconstruct individual viral haplotypes within patients with mixed infections. By reconstructing the full-length genome, we were able to pinpoint the likely timing, origins, and natural history of HCMV superinfections and uncover within-host viral recombination.

By providing a tool for viral haplotype reconstruction which is also suitable for DNA viruses with large genomes, we aim to simplify the investigation of mixed infections and within-host evolution for all viruses, both when longitudinal sequences are and are not available.

## Material and methods

HaROLD involves an initial estimation step followed by a refinement step. We describe the methods here. Further details are included in Supplementary Materials.

### Initial estimation

We start with a set of sequence reads from related samples that have been analysed using NGS. We initially assume that these samples contain a common set of haplotypes, but in differing proportions, an assumption that will be relaxed at a later stage.

We start with an assumed total number of haplotypes for the set of samples. Following quality control and assembly of the reads, for each sample, we count the number of each type of base observed at each position in the resulting alignment. The observed number of each base depends on (1) the frequencies of the haplotypes in that sample, (2) the base found at that position in each of the haplotypes, and (3) the probability of making an erroneous measurement at that site. As the error rate may be different at different sites and on different strands, we consider that this rate is drawn from a Dirichlet distribution. We first find the maximum likelihood estimate of the haplotype frequencies in each sample and the parameters defining the error rate distribution. We account for our initial ignorance of the haplotype sequences by summing this likelihood over all possible ways the different bases observed at that position can occur in the different haplotypes. We also integrate over the distribution of error rates.

Following estimation of the haplotype frequencies and error rate distribution parameters, we determine how much each assignment of bases to haplotypes contributes to the overall likelihood. This allows us to calculate the posterior probability of each assignment of bases to haplotypes. By summing over these posterior probabilities, we can compute the marginal posterior probability that a base is found at that site in each of the haplotypes. If these probabilities are sufficiently definitive, an assignment is made. The a posteriori marginal probability of each base is included in the output.

We perform this procedure for a range of different numbers of haplotypes. As increasing the number of haplotypes increases the number of ways of assigning bases to each of the haplotypes, decreasing the prior probability of any given assignment, the log likelihood typically decreases when the number of haplotypes increases beyond that necessary to represent the data. We select the number of haplotypes that maximise the log likelihood.

### Further refinement

The method described previously takes advantage of the presence of the same haplotype in multiple samples at various frequencies. It assumes that, although the haplotype sequences are described probabilistically, these probabilities are identical for all of the various samples, neglecting processes such as mutations. It also ignores the information that forms the basis of most haplotype reconstruction methods and the presence of multiple variants on the same read. The next step is to relax these assumptions and use variant co-localisation to refine the haplotypes.

In this refinement step (Fig. 1), each sample is analysed individually. We start with the estimated frequencies of each haplotype in this sample, and the a posteriori probability of each base at each site in each haplotype, as output from the previous program. The haplotypes are then optimised by assigning the reads, probabilistically, to the various haplotypes. The number of reads assigned to each haplotype is used to adjust the frequencies of each haplotype. The reads are then reassigned until the haplotype frequencies have converged. The resulting assigned reads are then used to update the probability of the bases found in each site in all of the reads assigned to each haplotype. This process is performed until convergence.

User-requested haplotype modifications are implemented. These include (1) recombination of two haplotypes, where corresponding regions of the haplotype sequences are swapped, (2) gene conversion, where a region of one haplotype sequence is overwritten by the corresponding region of a different haplotype sequence, (3) merging of two haplotypes into a single new haplotype, reducing the total number of haplotypes by one, and (4) dividing a single haplotype into two new haplotypes, increasing the total number of haplotypes by one.

First, the program considers an adjustable number of possible recombination of the haplotypes. These recombination events involve (1) picking two haplotypes at random, (2) picking a region of the alignment, of length chosen from a normal distribution with standard deviation of ten sites, and then (3) either swapping the values of the probabilities of the different bases in this region between the two haplotypes (50 per cent probability) or overwriting the values in one haplotype with the values from the other (25 per cent probability for each direction). Following such a step, the haplotype frequencies and base probabilities are then re-optimised as described previously, and the recombination event is either accepted or rejected based on whether the penalised log likelihood, that is, the log likelihood minus the number of adjustable parameters defining the haplotypes, is increased or decreased.

The program then implements an iterative process of refinement. At the start of each iteration, if requested, pairs of haplotypes are chosen and merged, with the frequencies of the resulting haplotype equal to the sum of that of the parents, and the base frequencies equal to the average of the two parents. This results in a reduction in the number of haplotypes by one. The haplotypes are then re-optimised. This process is repeated for every pair of haplotypes. The merge that most increases the penalised log likelihood is recorded.

If requested, a haplotype is chosen and split into two haplotypes, increasing the total number of haplotypes by one. The resulting set of haplotypes is then re-optimised. This is repeated for every original haplotype. The split that results in the largest increase in penalised log likelihood is recorded. Finally, the recombination process described previously is performed. Again, the recombination event that results in the largest increase in penalised log likelihood is recorded. Following these attempted modifications of the haplotypes, the modification—merge, split, or recombination—that most increases the penalised log likelihood is compared with the penalised log likelihood at the beginning of the iteration. If this results in a net increase in the penalised log likelihood, this modification is accepted and becomes the starting position for the next iteration. This iterative process is then repeated until convergence.

### Preparation of synthetic test datasets

The first four synthetic datasets consisted of mixtures of two to four norovirus sequences (approximately 7.5 kb in length) (Table 1); four additional datasets were assembled from two to three human cytomegalovirus (HCMV) sequences (approximately 230 kb) (Table 2). SimSeqNBProject (Benidt and Nettleton 2015) (https://github.com/jstjohn/SimSeq) was used to create 1,000,000 paired end reads of length 250 for each GenBank norovirus sequence listed in Table 1, and 100,000 paired end reads for each GenBank CMV sequence are listed in Table 2. SimSeq includes the getErrorProfile module which generates the error model for the sequence simulator. The output SAM files from SimSeq were then converted into Fastq files using Picard version 2.21.1 ‘SamToFastq’ (Broad Institute 2019). In order to construct the datasets, Seqtk 1.3 (https://github.com/shenwei356/seqkit) (Shen et al. 2016) was used to mix the reads from each ensemble according to the relative fractions listed in Tables 1 and 2. Reads were then trimmed for adapters using Trim galore version 0.6.0 (Krueger et al. 2019). Duplicate reads were removed using Picard version 2.21.1 ‘MarkDuplicates’. Reads were mapped to the GII.Pe-GII.4 Sydney 2012 reference strain JX459907 for norovirus and the Merlin reference strain NC_006273.2 for CMV using BWA version 0.7.17 (Li and Durbin 2009). The Makereadcount.jar (https://github.com/ucl-pathgenomics/HaROLD/tree/master/jar) was used to obtain the strand specific nucleotide counts from BAM files. These strand count files were used as the input for HaROLD.

### Evaluation of performance

We evaluated performance of haplotype reconstruction based on accuracy of reconstructed sequences and accuracy in reporting haplotype frequency or abundance in the sample. Accuracy of reconstructed sequences was calculated as SNPs differences between the GenBank sequences and the reconstructed haplotypes using the ‘dist.dna’ function in R library ‘ape’ (5.4–1) (Paradis and Schliep 2019) which produces a matrix of pairwise distances from DNA sequences. The model used was ‘raw’, simply the proportion of sites that differ between each pair of sequences. Frequency accuracy was calculated as a difference between the real haplotypes frequencies and frequencies calculated by the software (as 1–abs(real haplotype frequency—estimated haplotypes frequency)).

We estimated average heterozygosity (with an in-house script) for each sample as a measure of genetic diversity based on the reconstructed haplotypes.

### Comparison with other haplotype reconstruction programs

We compared HaROLD performance with the latest version of EVORhA, CliqueSNV, and PredictHaplo (v. 5). EVORhA was run with default parameters. We ran CliqueSNV with -tf option (minimum threshold for frequency relative to the read’s coverage) set to 0.01 (default was 0.05, decreasing the parameters increase the sensitivity of the program) and -cm option set as ‘fast’. PredictHaplo was run with default parameters as the HIV example included in the programs, except for entropy threshold, which was set to 0.05, max gap fraction to 0.05, local window size factor to 0.9, and Markov chain Monte Carlo interaction to 100, and deletions were not included.

### Validation with HCMV dataset (real data)

This patient was sequenced as part of the Wellcome Trust Collaborative Awards 204,870. The project includes HCMV sequencing from liver and kidney transplant recipients and donors. Seven samples were available for five time points for PatientA. All samples were mapped to Merlin GenBank sequence (NC_006273.2). The average read depth varied from 10x to 360x. Data were prepared for HaROLD in the same way as for the synthetic datasets. HaROLD analysis was run in the five samples from the initial run. Data were then aligned with Mafft (Katoh et al. 2002) and trees were obtained with Iqtree 1.6.12 (Nguyen et al. 2015) with GTR model and 500 bootstrap and plotted with Figtree (https://github.com/rambaut/figtree/releases/tag/v1.4.4).

### Validation on norovirus dataset (real data)

We have described this patient previously (Ruis et al. 2018). Fastq files were mapped to the closest GenBank reference (FJ537136) using the same pipeline used with the synthetic datasets. Haplotypes from HaROLD, EVORhA, CliqueSNV, and PredictHaplo were obtained similarly to the synthetic datasets. Data were then aligned with Mafft (Katoh et al. 2002) and trees were obtained with RAXML version 8 with GTRGAMMA model and 1,000 bootstraps (Stamatakis 2014). Pairwise distances were retrieved with ‘ape’ package in R (Paradis and Schliep 2019). Trees were plotted using ggtree (version 2.4.1) (Yu et al. 2017).

### R version and packages

Unless otherwise stated, all statistical analysis and plots were done in R 4.0.3, ggplot2 (3.3.5) (Wickham 2016), and ggpubr (0.4.0) (https://rpkgs.datanovia.com/ggpubr/index.html).

## Supplementary Material

veac093_Supp

## Data Availability

The software HaROLD and other materials used are available in the GitHub repository https://github.com/ucl-pathgenomics/HAROLD. Current release (v2.0) can be found in https://github.com/ucl-pathgenomics/HaROLD/releases/tag/v2.0.0. HaROLD supports multiple threads.
