# Supplementary material for: Haplotype assignment of longitudinal viral deep sequencing data using covariation of variant frequencies
Source: Virus Evol. 2022 Oct 6;8(2):veac093. doi: 10.1093/ve/veac093 (PMC9719071; doi:10.1093/ve/veac093)
Supplement: veac093_Supp [file veac093_supp.zip › suppl_data/NewSupp_LATEST.pdf]

SUPPLEMENTARY MATERIALS

DETAILS OF THE METHOD:

Initial estimation

The first step of the haplotype assignment method is designed to take advantage of the presence of multiple genomic samples, each containing a mixture of haplotypes; the set of haplotypes is assumed to be the same for every sample, although they are present in different proportions in the different samples. (The assumption of identical haplotypes is relaxed in the refinement step.) For the initial step, we start with an alignment containing all the reads mapped to the genome. The program receives as input the numbers of each base present at each site in the genome for each of the samples and generates a) a probabilistic representation of the haplotypes, b) the frequency of each haplotype in each sample, and c) the parameters describing the distribution of error rates.

The initial step of the haplotype analysis ignores the correlations between the bases observed at different sites along a single read. Instead, it matches a statistical model to base count data of the form $\{n_{b,l,d,s}\}$, representing the number of observed reads with base b at position l on a strand with direction d obtained from sample s. We consider the situation of longitudinal sampling, so that the observed reads for the different samples are assumed to come from a common set of H haplotypes $\{\overrightarrow{x_k}\}$ where $\overrightarrow{x_k} = \{x_{1,k}, x_{2,k}, x_{3,k} \dots x_{M,k}\}$ is the sequence of length M of haplotype k. The haplotypes are present in the different samples in different proportions: the frequencies of haplotypes in sample s is given by $\overrightarrow{\Phi_s} = \{\Phi_{1,s}, \Phi_{2,s}, \Phi_{3,s} \dots \Phi_{H,s}\}$ which obey $\sum_k \Phi_{k,s} = 1$. We wish to use $\{n_{b,l,d,s}\}$ to infer the haplotype sequences $\{\overrightarrow{x_k}\}$ and frequencies $\{\overrightarrow{\Phi_s}\}$.

Multiple haplotypes might share the same base at a given location; the expected frequency $\pi_{b_i,l,d,s}$ of base b_i at position l on a strand with direction d for sample s is equal to the sum of the frequencies of all haplotypes that have that base at that position: $\pi_{b_i,l,d,s} = \pi_{b_i,l,s} = \sum_{k\,\forall\, x_{l,k}=b_i} \Phi_{k,s}$ where we have written $\pi_{b_i,l,s} = \pi_{b_i,l,d,s}$ to indicate that the base frequencies are expected to be equal for the two strand directions.

The probability of observing a given base is affected by sequencing and mapping errors. Let us first consider the simple situation where there is a single fixed error rate ϵ. In such a situation, there is a probability $1 - 3\epsilon$ of observing the true base and a probability ϵ of observing one of the three other bases. If we know $\pi_{b_i,l,s}$, then the probability $P_{l,d,s}(b_i)$ of base b_i being observed, either correctly or erroneously, at position l on a read with the indicated strand direction and sample is given by $1 - 3\epsilon$ times the probability that base b_i is the true base plus ϵ times the probability that it is not the true base. (We include the strand direction in $P_{l,d,s}(b_i)$ to allow the error rates to be different for the different directions, as described below.)

$$
\begin{aligned}
P_{l,d,s}(b_i) &= \pi_{b_i,l,s}(1 - 3\,\epsilon) + \epsilon \sum_{b_j \neq b_i} \pi_{b_j,l,s} \\
&= \pi_{b_i,l,s} + \left(1 - 4\,\pi_{b_i,l,s}\right)\epsilon
\end{aligned}
\tag{1}
$$

where we have used the fact that $\sum_{b_j} \pi_{b_j,l,s} = 1$. The probability of observing $\{n_{b,l,d,s}\}_{l,d,s}$, the reads for a specified site, strand direction and sample, is then given by the multinomial distribution

$$P\left(\{n_{b,l,d,s}\}_{l,d,s}|\{\pi_{b,l,s}\},\epsilon\right) = \frac{n_{l,d,s}!}{\prod_{b_i} n_{b_i,l,d,s}!}\prod_{b_i} P_{l,d,s}(b_i)^{n_{b_i,l,d,s}}$$
$$= \frac{n_{l,d,s}!}{\prod_{b_i} n_{b_i,l,d,s}!}\prod_{b_i}\left(\pi_{b_i,l,s} + \left(1 - 4\,\pi_{b_i,l,s}\right)\epsilon\right)^{n_{b_i,l,d,s}} \quad (2)$$

where $n_{l,d,s} = \sum_{b_i} n_{b_i,l,d,s}$.

We do not know the values of ϵ, and cannot assume that the probabilities of misreads are the same for all real bases, observed bases, strand directions and samples. As we cannot estimate the error rate as a function of these specifics, we assume that the base- direction- and sample-specific error rate is drawn from a distribution. In this case, rather than being able to specify the probability $P_{l,d,s}(b_i)$ of observing each of the possible bases as in Equation (1), we can only specify the *distribution* of these probabilities, and then integrate over this distribution.

We do this by representing the expected distribution $\rho\left(\{P_{l,d,s}(b_i)\}|\{\pi_{b,l,s}\},\alpha_0,\alpha_\epsilon\right)$ of probabilities of observing any of the four bases $\{P_{l,d,s}(b_i)\}$ as a Dirichlet distribution $\mathrm{Dir}_{\{\alpha_{b,l,s}\}}\left(\{P_{l,d,s}(b_i)\}\right)$ given base frequencies $\{\pi_{b,l,s}\}$ and adjustable parameters α_0 and α_ϵ, given by

$$\rho\left(\{P_{l,d,s}(b_i)\}|\{\pi_{b,l,s}\},\alpha_0,\alpha_\epsilon\right) = \mathrm{Dir}_{\{\alpha_{b,l,s}\}}\left(\{P_{l,d,s}(b_i)\}\right)$$
$$= \frac{1}{\mathrm{B}\left(\{\alpha_{b,l,s}\}\right)}\prod_{b_i} P_{l,d,s}(b_i)^{\alpha_{b_i},l,s} \quad (3)$$

where $\alpha_{b_i,l,s} = \alpha_0\,\pi_{b_i,l,s} + \alpha_\epsilon$ and $\mathrm{B}(\{\theta_i\})$ is the multivariate beta function $\mathrm{B}(\{\theta_i\}) \equiv \frac{\Gamma(\theta_1)\Gamma(\theta_2)\ldots\Gamma(\theta_n)}{\Gamma(\theta_1+\theta_2+\cdots+\theta_n)}$. For such a Dirichlet distribution, the average probability of observing a given base $\langle P_{l,d,s}(b_i)\rangle$ is given by

$$\langle P_{l,d,s}(b_i)\rangle = \frac{\alpha_0\,\pi_{b_i,l,s} + \alpha_\epsilon}{\alpha_0 + 4\alpha_\epsilon} = \pi_{b_i,l,s} + \left(1 - 4\,\pi_{b_i,l,s}\right)\left(\frac{\alpha_\epsilon}{\alpha_0 + 4\alpha_\epsilon}\right) \quad (4)$$

which mirrors Equation (1) when $\epsilon = \frac{\alpha_\epsilon}{\alpha_0+4\alpha_\epsilon}$.

This approach has two advantages. Firstly, we are not specifying an error rate but allowing the error rate to vary by an amount determined by the parameters in the Dirichlet distribution: the relative values of α_ϵ and α_0 determine the average error rate while the absolute magnitudes characterise its variation. Secondly, we do not need to make judgements about which bases are erroneous and which ones are not. Rather we represent the probability of observing a base, either correctly or erroneously, rather than representing the probability that the base actually exists at that site. This allows us to formulate the problem in terms of quantities that are directly observed rather than inferred. Although we assume that the distribution is symmetric with respect to the various bases, the rates for specific errors need not be the same.

We now have a distribution of $\{P_{l,d,s}(b_i)\}$ rather than specific values, so to calculate the likelihood $\Lambda\left(\{n_{b,l,d,s}\}_{l,d,s}|\{\overrightarrow{x_k}\}_l,\{\Phi_{k,s}\},\alpha_0,\alpha_\epsilon\right)$ of the observed data at that position and strand direction in the sample given we need to integrate over this distribution

$$\Lambda\left(\{n_{b,l,d,s}\}_{l,d,s}|\{\overrightarrow{x_k}\}_l,\{\Phi_{k,s}\},\alpha_0,\alpha_\epsilon\right) = \frac{n_{l,d,s}!}{\prod_{b_i} n_{b_i,l,d,s}!}\int \mathrm{Dir}_{\{\alpha_{b,l,d,s}\}}\left(\{P_{l,d,s}(b_i)\}\right)\prod_{b_i} P_{l,d,s}(b_i)^{n_{b_i,l,d,s}}\ d\left(\{P_{l,d,s}(b_i)\}\right) \quad (5)$$

where the integral is over the entire range of the Dirichlet distribution, which is all values of $\{P_{l,d,s}(b_i)\}$ such that $\sum_i P_{l,d,s}(b_i) = 1$. Note that as $\alpha_{b_i,l,s} = \alpha_0\ \pi_{b_i,l,s} + \alpha_\epsilon = \alpha_0\left(\sum_{k\ \forall\ x_{k,l}=b}\Phi_{k,s}\right) + \alpha_\epsilon$, the likelihood depends explicitly on the quantities of interest, including the bases $\{\overrightarrow{x_k}\}_l$ present in the set of haplotype sequences at position l as well as the haplotype frequencies $\{\Phi_{k,s}\}$ and the parameters α_0 and α_ϵ. Taking advantage of the properties of the Dirichlet distribution, this integral is straightforward, resulting in

$$\Lambda\left(\{n_{b,l,d,s}\}_{l,d,s}|\{\overrightarrow{x_k}\}_l,\{\Phi_{k,s}\},\alpha_0,\alpha_\epsilon\right) = \frac{n_{l,d,s}!}{\prod_{b_i} n_{b_i,l,d,s}!}\ \frac{\mathrm{B}\left(\{\alpha_0\ \pi_{b_i,l,s} + \alpha_\epsilon + n_{b_i,l,d,s}\}\right)}{\mathrm{B}\left(\{\alpha_0\ \pi_{b_i,l,s} + \alpha_\epsilon\}\right)} \quad (6)$$

The likelihood of the entire data set consisting of $\{n_{b,l,d,s}\}$, the number of times we observe each base at each location in the alignment in the reads with each directionality d in each sample s is then equal to

$$\Lambda\left(\{n_{b,l,d,s}\}|\{\overrightarrow{x_k}\},\{\Phi_{k,s}\},\alpha_0,\alpha_\epsilon\right) = \prod_l\prod_{d,s}\Lambda\left(\{n_{b,l,d,s}\}_{l,d,s}|\{\overrightarrow{x_k}\}_l,\{\Phi_{k,s}\},\alpha_0,\alpha_\epsilon\right) \quad (7)$$

As described above, we do not know the haplotype sequences $\{\overrightarrow{x_k}\}$. As this information is unavailable we calculate the likelihood by doing an exhaustive sum over all 4^H possible ways of assigning 4 bases to H haplotypes at each position. All such assignments are considered equally likely, with a priori probabilities 4^{-H}. The haplotype sequence is assumed to be the same for both strand directions and all samples; therefore, the sum over possible haplotype sequences must be performed outside the product over strand directions and samples, but we can consider each site separately.

$$\Lambda\left(\{n_{b,l,d,s}\}|\{\Phi_{k,s}\},\alpha_0,\alpha_\epsilon\right) = \prod_l\sum_{\{\overrightarrow{x_k}\}_l}\frac{1}{4^H}\prod_{d,s}\Lambda\left(\{n_{b,l,d,s}\}_{l,d,s}|\{\overrightarrow{x_k}\}_l,\{\Phi_{k,s}\},\alpha_0,\alpha_\epsilon\right) \quad (8)$$

As an example, if we have three haplotypes, so that the bases found in position l of the haplotypes is given by $\{\overrightarrow{x_k}\}_l = \{x_{l,1},x_{l,2},x_{l,3}\}$,

$$\Lambda\left(\{n_{b,l,d,s}\}|\{\Phi_{k,s}\},\alpha_0,\alpha_\epsilon\right) = \prod_l \frac{1}{64}\prod_{d,s}\begin{pmatrix}\Lambda(\{n_{b,l,d,s}\}|\{\mathrm{A,A,A}\},\{\Phi_{k,s}\},\alpha_0,\alpha_\epsilon) \\ +\Lambda(\{n_{b,l,d,s}\}|\{\mathrm{A,A,C}\},\{\Phi_{k,s}\},\alpha_0,\alpha_\epsilon) \\ +\Lambda(\{n_{b,l,d,s}\}|\{\mathrm{A,A,G}\},\{\Phi_{k,s}\},\alpha_0,\alpha_\epsilon) \\ \ldots+\Lambda(\{n_{b,l,d,s}\}|\{\mathrm{T,T,T}\},\{\Phi_{k,s}\},\alpha_0,\alpha_\epsilon)\end{pmatrix} \tag{9}$$

In practice, this sum is not overly large for the number of haplotypes typically supported by the data.

We first maximise this expression by adjusting the values of $\{\Phi_{k,s}\}$, α_0, and α_ϵ, noting that $\{\Phi_{k,s}\}$ is the same for all locations at each sample. We do this by choosing values for α_0, and α_ϵ, and then adjusting the $H \times S$ haplotype frequencies, where H is the number of haplotypes and S is the number of samples. Following optimisation of the haplotype frequencies, we optimise the parameters α_0, and α_ϵ defining the error rate distribution and iterate.

Up until this point the haplotype sequences are undefined. As all sets of bases assigned to the haplotypes at a given location are a priori equally likely, we can estimate the posterior probability of each assignment of bases to haplotypes $\{\overrightarrow{x_k}\}_l$ by seeing how much each contributes to the likelihood.

$$p(\{\overrightarrow{x_k}\}_l) = \frac{\prod_{d,s}\Lambda\left(\{n_{b,l,d,s}\}_{l,d,s}|\{\overrightarrow{x_k}\}_l,\{\Phi_{k,s}\},\alpha_0,\alpha_\epsilon\right)}{\sum_{\{\overrightarrow{x_j}\}_l}\prod_{d,s}\Lambda\left(\{n_{b,l,d,s}\}_{l,d,s}|\{\overrightarrow{x_k}\}_l,\{\Phi_{k,s}\},\alpha_0,\alpha_\epsilon\right)} \tag{10}$$

By summing $p(\{\overrightarrow{x_k}\}_l)$ over all of the assignments with a given base at a site in a particular haplotype, we can obtain the probability $h_{l,k}(b_i)$ of base b_i at position l in haplotype k

$$h_{l,k}(b_i) = \sum_{\{\overrightarrow{x_k}\}_l \,\forall\, x_{l,k}=b_i} p(\{\overrightarrow{x_k}\}_l) \tag{11}$$

These probabilities are provided by the program, as well as explicit haplotype sequences where the probability of a given base is over a user-defined threshold.

Choosing the number of haplotypes

This initial stage assumes a fixed, pre-specified number of haplotypes, that is the same for all samples. This procedure can be performed with a range of different haplotype numbers, and the number of haplotypes determined by which produces the maximum log likelihood.

For optimisation procedures, more complicated models will, in general, result in an increase in the fit to the data. When comparing models in such cases, there is a need to penalise more complicated models in order to prevent overfitting, as is described in the refinement process below. Bayesian methods typically are more robust to such problems. In this specific situation, the more haplotypes that are considered, the larger the number of possible ways of assigning bases to haplotypes, the lower the a priori probability of each assignment, including the assignments that best explain the observed data. It is this corresponding decline in the prior probabilities as the total probability (1) is divided by the number of possibilities that result in the decrease in the likelihood with increasing numbers of haplotypes. The difference is between optimisation of the haplotypes, where increasing the possibilities can only help, and summing over all possible haplotypes, where this summation is over larger and larger dimensionalities as the number of haplotypes is increased.

Refinement

As described above, the initial estimation discards any information about co-localisation of different variants on the same read, while assuming that the haplotype sequences in each sample are identical. In the refinement process we include co-localisation information, allowing us to relax the assumption of identical haplotypes.

The overall procedure is summarised in Figure 7. We start the refinement with estimated values of $h_{l,k}(b_i)$, the posterior probabilities that base b_i would be found in position l in haplotype k, and the frequency of each haplotype in sample s, $\{\Phi_{k,s}\}$, as provided by the initial estimation. These values can be considered as defining the set of haplotypes contained in the model.

Adjustment of haplotype frequencies and sequences

For the refinement procedure, we consider each sample separately, meaning that the set of haplotypes for the different samples are not identical. We consider the database consisting of reads $\{B_{l,r}\}$ with $B_{l,r}$ equal to the base observed at alignment position l in read r. (As we are doing each sample independently, we do not have to specify the sample, and we will drop the s subscript. $B_{l,r} = \emptyset$ if the read does not overlap position l.) Given the haplotype frequencies and the probabilistic haplotype sequence reconstructions, the joint probability that a read would be from haplotype k and would result in the observed sequence of read r is $\Phi_k \prod_l h_{l,k}(B_{l,r})$; the total probability of read r being produced by some haplotype is just this summed over all haplotypes, $\sum_{k'}(\Phi_k \prod_l h_{l,k}(B_{l,r}))$. Following Bayes' theorem, we calculate the probability $P_r(k)$ that read r belongs to haplotype k as

$$P_r(k) = \frac{\Phi_k \prod_l h_{l,k}(B_{l,r})}{\sum_{k'}(\Phi_{k'} \prod_l h_{l,k'}(B_{l,r}))} \quad (12)$$

The log likelihood of the set of observed reads is just equal to the sum of the log likelihood of each individual read

$$\log \Lambda(\{B_{l,r}\}) = \sum_r \log \sum_{k'} \left(\Phi_{k'} \prod_l h_{l,k'}(B_{l,r}) \right) \quad (13)$$

We distribute the contribution of each read amongst the haplotypes according to the probabilities calculated in (12). Summing up these contributions for each haplotype yields the expected number of reads coming from that haplotype. Distinguishing these contributions by the base in the read at position l provides the distribution of bases observed at that position. This allows us to re-estimate Φ_k and $h_{l,k}(B_{l,r})$. Note that, as $P_r(k)$ is an explicit function of Φ_k and $h_{l,k}(B_{l,r})$, re-estimation of these parameters will change the probabilistic distribution of reads amongst the various haplotypes, requiring a further re-estimation of these parameters. We first update Φ_k

$$\Phi'_k = \frac{\sum_r P_r(k)}{R} \quad (14)$$

where R is the total number of reads. These updated haplotype frequencies are then used to re-assign reads to haplotypes using Equation (12), followed by re-estimation of Φ'_k with Equation(14); this process is repeated until there is a negligible change in the log likelihood as calculated with Equation (13).

We next update the haplotype observed base frequencies $h_{l,k}(b_i)$ by a similar method, considering the bases of the haplotypes that have been probabilistically assigned to each of the haplotypes:

$$h'_{l,k}(b_i) = \frac{\sum_{r \,\forall\, b_i = B_{l,r}} P_r(j)}{\sum_r P_r(j)} \tag{15}$$

Again, we iteratively use these new observed base frequencies to re-assign reads to haplotypes using Equation (12), followed by re-estimation of $h_{l,k}(b_i)$ with Equation (15), until there is a negligible change in the log likelihood.

This process of adjusting Φ_k and then $h_{l,k}(B_{l,r})$ is itself repeated until convergence, again measured by the resulting change in the log likelihood.

Recombination

In order to accelerate convergence, we also consider larger-scale changes in the set of haplotypes defined by $h_{l,k}(B_{l,r})$. In particular, we use a recombination move to escape from local optima where contiguous stretches of a specific haplotype in the model correspond to different haplotypes in the sample. Two haplotypes are chosen at random, and we select a random segment of the sequences with a length chosen from a folded Normal distribution of mean 0 and standard deviation 10. We then either exchange the values of $h_{l,k}(B_{l,r})$ between the two haplotypes for sites in this segment (probability 50%) or over-write the values of $h_{l,k}(B_{l,r})$ for one haplotype with the values for the other (probability 50%). Note that this procedure is motivated by computational efficiency and is not designed to mirror the biological process of recombination.

Following this recombination step, we re-adjust Φ_k and then $h_{l,k}(B_{l,r})$ as described above. We then compare the log likelihood of the data (Equation (13)) before and after the recombination, only accepting the recombination step if the log likelihood increases. In the absence of such an increase, the parameters defining the set of haplotypes is reset to the pre-recombination values.

Adjusting the number of haplotypes

The initial stage of the calculation is performed with the number of haplotypes as an input. This stage is performed with a range of different haplotype numbers, and the appropriate number of haplotypes would be equal to the number that provides the highest log likelihood. For the refinement process, we can allow this number to vary by dividing haplotypes, increasing the number of haplotypes by one, or combining haplotypes, decreasing the number of haplotypes by one. We use a penalised log likelihood function to determine how many haplotypes can be justified based on the read data. The penalised log likelihood is equal to the log likelihood minus the number of adjustable parameters, P equal to -0.5 times the Akaike Information Criterion (AIC).

Calculating the number of parameters in the model is not obvious. The simplest approach would be to count the entire set of values of $h_{l,k}(B_{l,r})$, equal to $3MH$, where M is the length of the genome, H is the number of haplotypes, and 3 reflects the number of independent values of $h_{l,k}(B_{l,r})$ for the four bases constrained to $\sum_{b_i} h_{l,k}(b_i) = 1$; plus the $H-1$ independent values of Φ_k, constrained by $\sum_k \Phi_k = 1$. We note, however, that the evidence in support of a given number of haplotypes would seem not to be lessened by the addition of a large number of perfectly conserved sites in the genome, although this would result in a large increase in M. More generally, we could argue that a site with only two observed bases represents a constraint on all of the values of $h_{l,k}(B_{l,r})$ for that site, that the value of this parameter is zero for non-observed bases. In this case, we would consider only the parameters necessary to characterise the haplotypes given the bases that are actually observed. For these reasons, we use $\mathrm{P} = H\sum_l \left(m_l^{obs} - 1\right) + H - 1$ where m_l^{obs} is the number of different bases observed at that site, with $1 \le m_l^{obs} \le 4$. We recognise that this is an area of controversy, and ultimately the choice is made heuristically based on a balance between computational and conceptual simplicity versus degree of detail.

Aside from the use of penalised log likelihood, the procedure is similar to that for recombination. For a merge, two haplotypes are chosen, and a new haplotype is created based on the statistics of the union of the reads probabilistically assigned to the two haplotypes; the two original haplotypes are then deleted. We then re-adjust Φ_k and then $h_{l,k}(B_{l,r})$ as described above. We then compare the penalised log likelihood of the data before and after the merge, considering the change in the number of adjustable parameters, and only accepting the result of the merge if the penalised log likelihood increases. In the absence of such an increase, the parameters defining the set of haplotypes is reset to the pre-merge values. This is done for all pairs of haplotypes in turn.

Splits of haplotypes are performed in a similar manner. A haplotype is chosen and used to create two new haplotypes. If the haplotype has a consensus base at a location, that consensus base is also used for the new haplotypes. If the haplotype is variable at a given site, one haplotype consistently receives the most frequent base whilst the other consistently receives the least frequent base. The original haplotype is then deleted. Once again we re-adjust Φ_k and then $h_{l,k}(B_{l,r})$ as described above, and accept or reject the split based on whether the re-adjusted haplotypes have an increased penalised log likelihood. This is done for all original haplotypes in turn.

These processes of recombination, merging, and splitting, combined with the readjustment of model parameters, is performed until we observe no significant increase in the penalised log likelihood, at which time the analysis is halted and the output files are written.

Supplementary figures

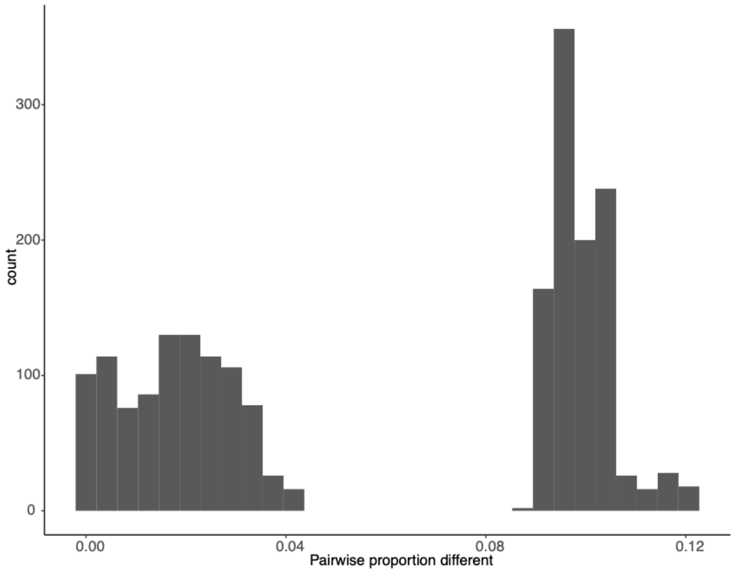

Supplementary figure 1: Pairwise genetic distances calculated as proportion. Genetic distances were calculated for all reconstructed haplotypes obtained with HaROLD from 12 norovirus samples from an immunocompromised patient.

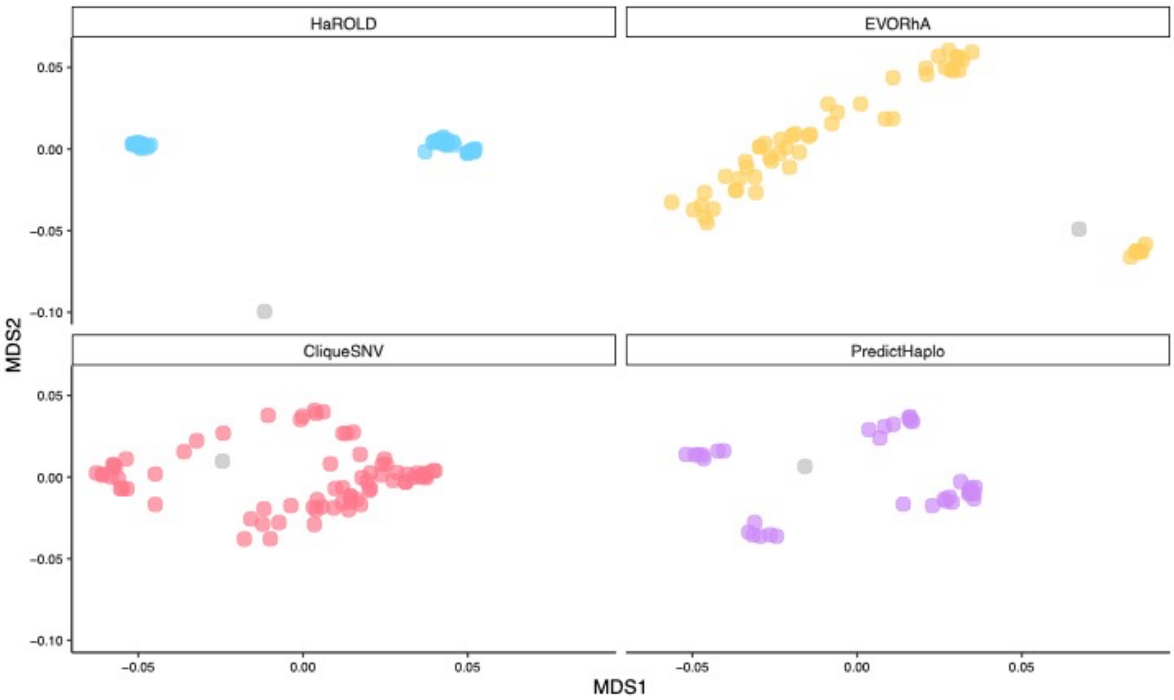

Supplementary figure 2: multi-dimensional scaling (MDS) of reconstructed haplotypes for patient infected with norovirus for different methods. Pairwise differences between haplotypes were calculated and used for MDS clustering. X-axis shows the first component obtained with MDS (MDS1) plotted against the second MDS component (y-axis, MDS2). Reference GenBank strain is coloured in grey.

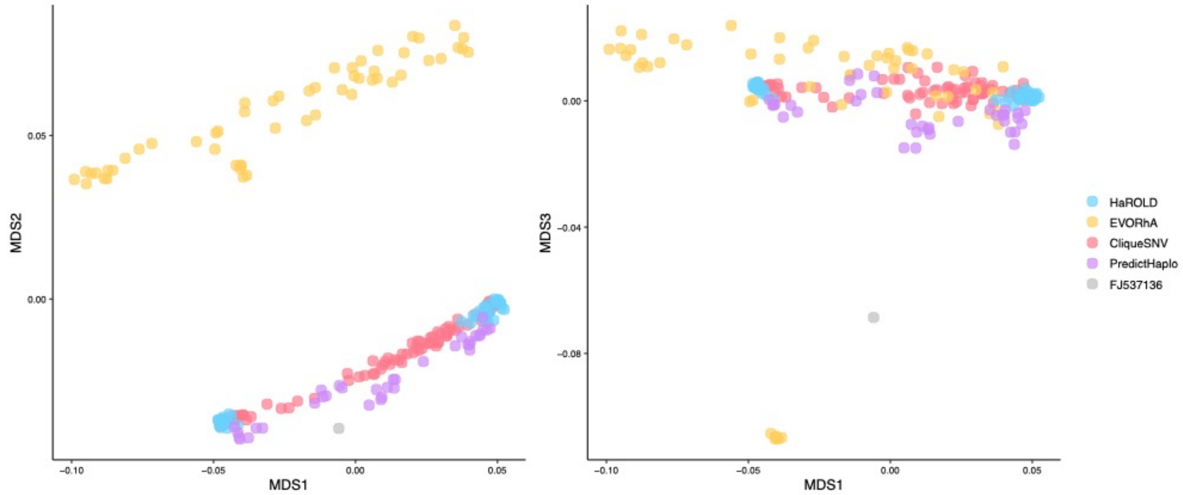

Supplementary figure 3: multi-dimensional scaling (MDS) for all sequences obtained with different methods for patient infected with norovirus. All haplotypes obtained were aligned together to analyse the relationship between sequences retrieved by different methods. Pairwise differences between haplotypes were calculated and used for MDS clustering. Left plot shows the first component obtained with MDS (X-axis, MDS1) plotted against the second MDS component (y-axis, MDS2). Right plot shows the first component obtained with MDS (X-axis, MDS1) plotted against the third MDS component (y-axis, MDS3). Reference GenBank strain is coloured in grey.

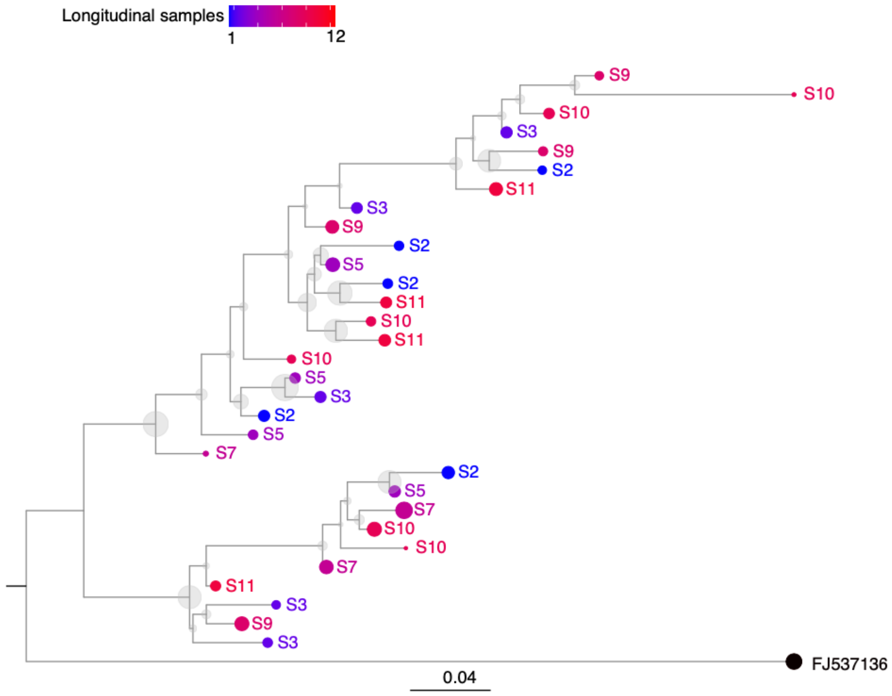

Supplementary figure 4: Maximum likelihood phylogenetic tree of PredictHaplo haplotypes reconstructed haplotypes for patient infected with norovirus. Twelve samples were available for this patient (S1-S12) and were coloured differently using a continuous scale representing time (from blue S1 to red S12). The tips' size indicates the frequency of the haplotype. The black sequence is the Genbank strain used for mapping (tip size set as 50% frequency). Grey transparent circles represent the bootstrap values (1000 bootstraps in total).

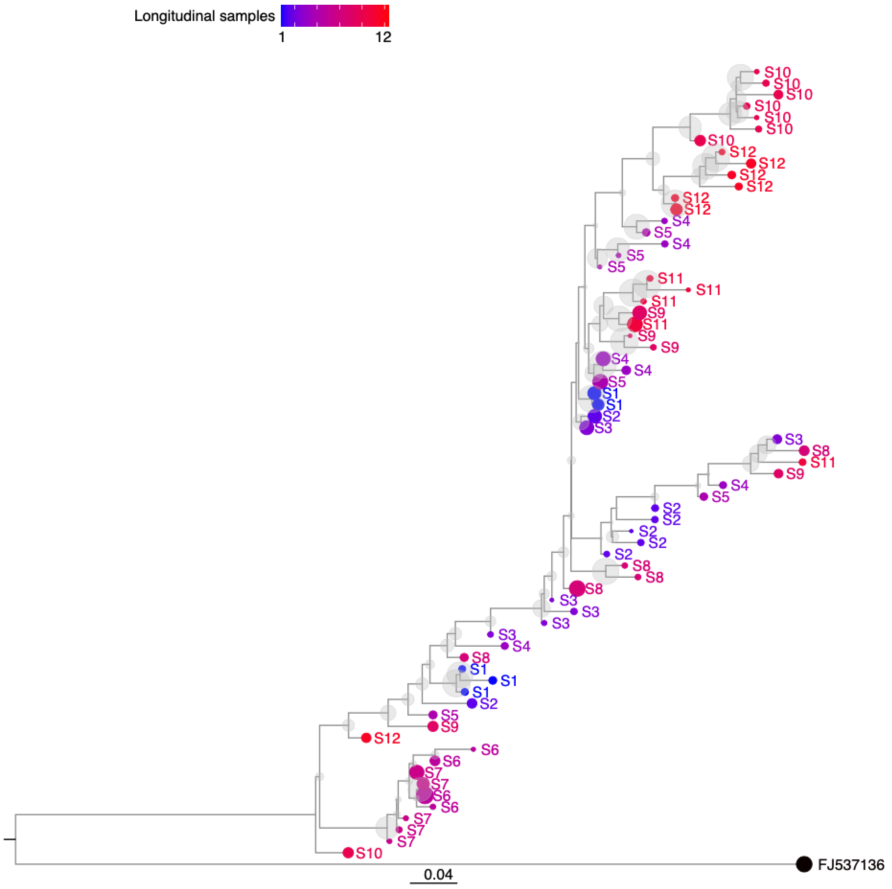

Supplementary figure 5: Maximum likelihood phylogenetic tree of CliqueSNV haplotypes reconstructed haplotypes for patient infected with norovirus. Twelve samples were available for this patient (S1-S12) and were coloured differently using a continuous scale representing time (from blue S1 to red S12). The tips' size indicates the frequency of the haplotype. The black sequence is the Genbank strain used for mapping (tip size set as 50% frequency). Grey transparent circles represent the bootstrap values (1000 bootstraps in total).

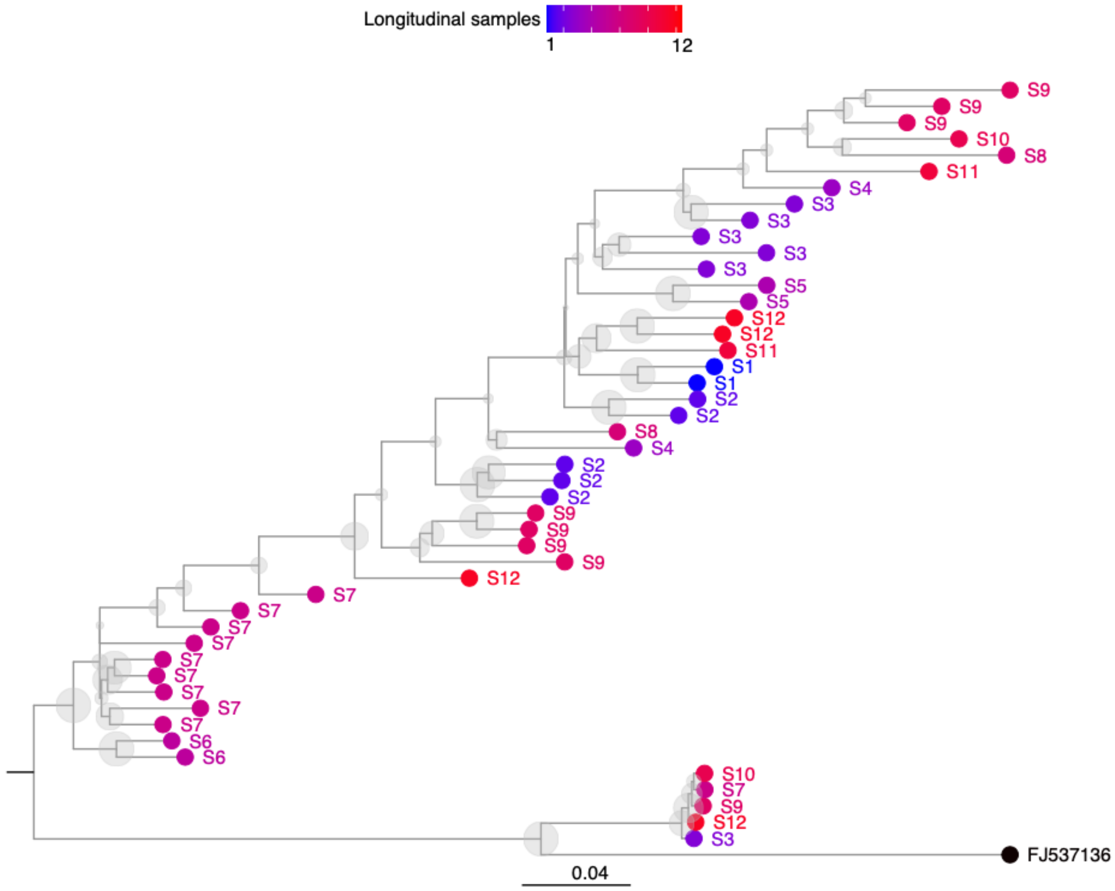

Supplementary figure 6: Maximum likelihood phylogenetic tree of EVORhA haplotypes reconstructed haplotypes for patient infected with norovirus. Twelve samples were available for this patient (S1-S12) and were coloured differently using a continuous scale representing time (from blue S1 to red S12). Haplotypes' frequency was always <1%, for this reason tips' size in the plot are all set at the same size here. The black sequence is the GenBank strain used for mapping (tip size set as 50% frequency). Grey transparent circles represent the bootstrap values (1000 bootstraps in total).
